# A Tablet App– and Sensor-Based Assistive Technology Intervention for Informal Caregivers to Manage the Challenging Behavior of People With Dementia (the insideDEM Study): Protocol for a Feasibility Study

**DOI:** 10.2196/11630

**Published:** 2019-02-26

**Authors:** Sven Kernebeck, Daniela Holle, Patrick Pogscheba, Felix Jordan, Fabian Mertl, Alina Huldtgren, Sebastian Bader, Thomas Kirste, Stefan Teipel, Bernhard Holle, Margareta Halek

**Affiliations:** 1 German Center for Neurodegenerative Diseases Witten Germany; 2 Faculty of Health University of Witten/Herdecke Witten Germany; 3 Faculty of Media Hochschule Düsseldorf University of Applied Sciences Düsseldorf Germany; 4 Institute of Computer Science University of Rostock Rostock Germany; 5 German Center for Neurodegenerative Diseases Rostock/Greifswald Germany

**Keywords:** dementia, technology, caregivers, telemedicine, program evaluation, interventions, behavioral symptoms

## Abstract

**Background:**

Despite the enormous number of assistive technologies (ATs) in dementia care, the management of challenging behavior (CB) of persons with dementia (PwD) by informal caregivers in home care is widely disregarded. The first-line strategy to manage CB is to support the understanding of the underlying causes of CB to formulate individualized nonpharmacological interventions. App- and sensor-based approaches combining multimodal sensors (actimetry and other modalities) and caregiver information are innovative ways to support the understanding of CB for family caregivers.

**Objective:**

The main aim of this study is to describe the design of a feasibility study consisting of an outcome and a process evaluation of a newly developed app- and sensor-based intervention to manage CB of PwD for family caregivers at home.

**Methods:**

In this feasibility study, we perform an outcome and a process evaluation with a pre-post descriptive design over an 8-week intervention period. The Medical Research Council framework guides the design of this feasibility study. The data on 20 dyads (primary caregiver and PwD) are gathered through standardized questionnaires, protocols, and log files as well as semistructured qualitative interviews. The outcome measures (neuropsychiatric inventory and Cohen-Mansfield agitation inventory) are analyzed by using descriptive statistics and statistical tests relevant to the individual assessments (eg, chi-square test and Wilcoxon signed-rank test). For the analysis of the process data, the Unified Theory of Acceptance and Use of Technology is used. Log files are analyzed by using descriptive statistics, protocols are analyzed by using documentary analysis, and semistructured interviews are analyzed deductively using content analysis.

**Results:**

The newly developed app- and sensor-based AT has been developed and was evaluated until July in 2018. The recruitment of dyads started in September 2017 and was concluded in March 2018. The data collection was completed at the end of July 2018.

**Conclusions:**

This study presents the protocol of the first feasibility study to encompass an outcome and process evaluation to assess a complex app- and sensor-based AT combining multimodal actimetry sensors for informal caregivers to manage CB. The feasibility study will provide in-depth information about the study procedure and on how to optimize the design of the intervention and its delivery.

**International Registered Report Identifier (IRRID):**

DERR1-10.2196/11630

## Introduction

### Background

The management of dementia is complicated by the presence of behavioral and psychological symptoms, also referred to as challenging behavior (CB) [1,2]. CB includes a wide range of behaviors such as screaming, restlessness, wandering, pilfering, or hoarding [3]. CB represents a complex construct that results from the interaction of biological, psychological, and social factors that are idiosyncratic to the person with dementia (PwD) [4]. This behavior causes considerable stress for family caregivers [5] and is one of the most common reasons why family members transfer care responsibilities to residential care, for example, nursing homes [6,7]. Due to the limited positive effects of psychotropic medications and their tremendous adverse effects [8,9], individualized nonpharmacological approaches combining caregiver education and support with direct intervention for the PwD are the first-line strategies to manage CB [10,11]. Consequently, the current guidelines on CB emphasize the importance of describing the behavior and the context in which behavioral symptoms occur as well as identifying potential modifiable triggers for CB from which to derive a treatment plan to address the underlying contributors [12-14]. Therefore, approaches are needed that include an assessment of the topography (nature, duration, and frequency), consequences (safety and stress), and multitude of the possible bio-psycho-social causes of CB. Afterwards, the results of the assessment must be linked to individual interventions in a meaningful way [15]. To date, systematic approaches incorporating both the description of the behavior and its underlying causes and linking the assessment to individualized interventions in a meaningful way, especially for the homecare environment, are rare [15].

Several widely used instruments are available to assess CB such as the neuropsychiatric inventory (NPI) [16], the Cohen-Mansfield agitation inventory (CMAI) [17], and in homecare, the revised memory and behavior problems checklist [18]. However, the primary focus of these instruments is the description of the behavior rather than the understanding of the underlying causes of CB [19]. In the German context, the *Innovative dementia-oriented Assessment system (IdA)* is available, which was originally developed to systematically guide nursing staff in the description and analysis of the underlying causes of CB of nursing home residents [19]. The theoretical framework of the IdA instrument is the need-driven dementia-compromised behavior model [20]. There is evidence that the IdA instrument in combination with dementia-specific case conferences stimulates self-reflection and external reflection about the CB by members of nursing staff [21]. Furthermore, this approach supports the nursing staff to describe the CB and its circumstances more accurately [21]. Although the IdA instrument was originally developed for use in the nursing home setting, it might also be a useful instrument for family caregivers in the home care setting. To support family caregivers in the caring of PwD and the management of CB, many different approaches have been developed [1]. In this regard, many studies have highlighted the potential of assistive technologies (ATs) to support family caregivers [22]. AT is an umbrella term that describes “a product, equipment or device, usually electronic or mechanical in nature, which helps people with disabilities to maintain their independence or improve their quality of life” [23,24]. Despite the enormous number and diversity of ATs in dementia care [24], the technology-based management of CB is highly underrepresented [25,26]. ATs combined with multimodal actimetry sensor technology might provide a promising and innovative addition to the existing face-to-face approaches for family caregivers [27,28]. Actimetry sensors can capture wide facets of CB by measuring acceleration, movement, rotation, and the location of an individual. In-depth information about the context in which the CB occurs can be assessed particularly well by measuring air pressure, loudness, and light level with actimetry sensors [29]. Although using standardized assessments, these reports are related to the point of view of the caregiver, which is influenced by many different factors. These factors can include the subjective view of the caregiver, the period that the caregiver and PwD spend together, or even the memory of the caregiver [30]. In addition, accelerometric measures show associations between the accelerometric motion score (AMS) and the physical nonaggressive behavior domain of CMAI [28]. To the best of our knowledge, there is only 1 Web-based technology, the WeCareAdvisor, that supports caregivers in analyzing the underlying causes and management of CB [31]; however, it does not include the potential to employ actimetry sensor technologies. The insideDEM study aims to develop and test the feasibility of an assistive technology–based intervention that includes a multimodal actimetry sensor technology for family caregivers of PwD to understand CB and to manage CB in the home care environment. The purpose of this paper is to describe the design of a feasibility study as the Medical Research Council (MRC) framework recommends in the development of complex interventions. The feasibility study includes outcome and process evaluations.

### Objectives

The primary aim of the outcome evaluation is to test the study procedure and the practicability of the intervention itself and to select the appropriate outcomes. The main aim of the process evaluation is to gain information about the processes of delivery, the acceptance of the intervention, and the requirements to optimize the design of the intervention. Both evaluations contribute to the development of a pilot study and even a trial on a larger scale [32].

**Table 1:** Domains according to the Medical Research Council framework for process evaluation and research questions.

Domain	Subdomain	Research questions
A: Implementation of the intervention	Recruitment and reach of households	How were the households recruited for the intervention, and which individuals received the intervention?
	Delivery of the intervention to households	Was the intervention delivered as intended for each of the households?
	Adaptations of the implementation	What adaptations of the delivery of the intervention are made during the intervention phase?
B: Mechanism of impact of the intervention	Response or acceptance	How is the acceptance of the caregivers with respect to the intervention?
	Response or acceptance	How is the acceptance of persons with dementia with respect to the intervention?
	Unexpected consequences and pathways	Does the intervention lead to unexpected consequences and pathways?
	Adaptation of the intervention	What adaptations of the intervention had to be made during the intervention?
C: Context of the intervention	Ethical evaluation	What are the ethical implications of the assistive technology, and how are they influencing the design of the ATI^a^?
	Social and legal implications	What are the legal and social implications of the ATI in the German context regarding reimbursement by the statutory health insurance and the regulations of the German Act on Medical Devices (Medizinproduktegesetz)?
	Economic evaluation	What are the economic implications of the actual effort of the development, delivery and standard operating costs, and further costs for realizing a sufficient ATI?

^a^ATI: assistive technology intervention.

### Research Questions

The outcome evaluation is guided by the following research questions:

1. What effect does the assistive technology intervention (ATI) have on CB and agitation of the PwD?
2. What effect does the new ATI have on the (1) primary caregivers’ skills to manage the CB of the PwD, (2) quality of the current caregiving relationship to the PwD, (3) behavior-related distress, (4) self-perceived health, and (5) goals of caregiving?

The process evaluation is guided by the following research questions, which are subdivided into 3 domains (Table 1).

## Methods

### Study Design and Setting

This prospective exploratory feasibility study is a phase 2 study according to the MRC framework for the development and evaluation of complex health care interventions [32,33]. This study uses a pre-post design with an 8-week intervention period, without a control group. The setting of the study is the home environment of family caregivers and PwD in the region of Krefeld, North Rhine-Westfalia (Germany).

### Eligibility Criteria

#### Person With Dementia

A PwD is included in the study if he or she (1) has either a documented diagnosis of dementia or a Mini-Mental State Examination [34] score of 24 or less and (2) shows at least one CB according to NPI [35]. The exclusion criteria are a documented restless legs syndrome (International Statistical Classification of Diseases and Related Health Problems, ICD 10, G25.81), a Korsakoff syndrome (ICD 10, F10.6 and F11-F 19), or a disorder of adult personality and behavior (ICD 10, F60.0-F60.9).

#### Family Caregiver

A caregiver is included if he or she (1) is the primary caregiver; (2) lives in the same household as the PwD; (3) provides at least 4 hours/day of care; (4) understands, reads, and writes in the German language; (5) has no visual impairment; and (6) is willing to use the technology over the course of the intervention period. Specific competences in the use of any technology are not required.

### Intervention

**Figure 1:**
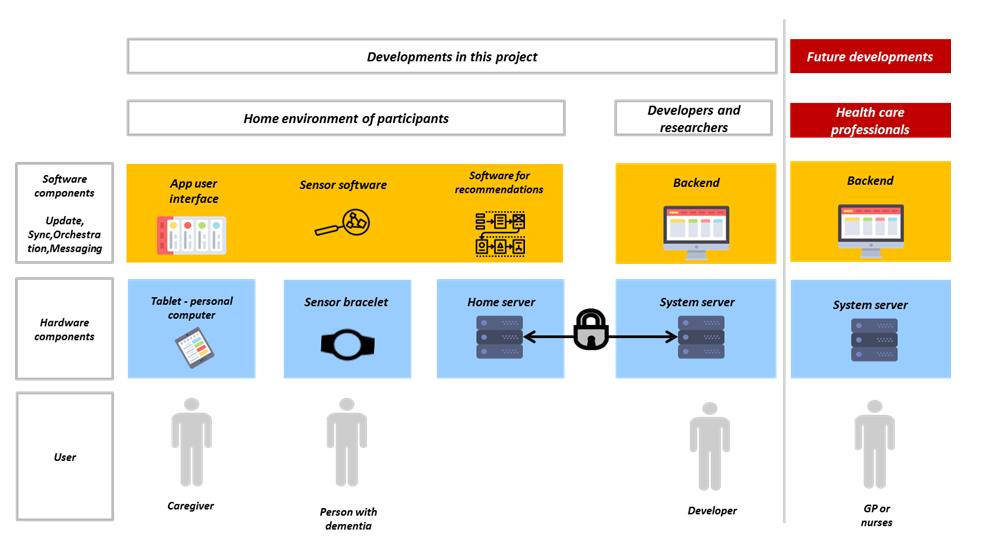
Software and hardware components of the insideDEM intervention. GP: general practitioner.

The new complex ATI for caregivers to manage the CB of PwD was developed by a multidisciplinary team using a user-centered design process with different methods: user workshops, usability tests, cognitive debriefing, and consecutive expert panels. The user of the ATI is a primary caregiver of a PwD. The ATI will be placed in the homes of caregivers of PwD, and it aims to support the caregiver in understanding the behavior of his or her family member with dementia, in monitoring their behavior, and in choosing individualized interventions. Moreover, the ATI should help the caregiver to collect and communicate information regarding behavior to relevant health care workers. The ATI consists of different hardware and software components (Figure 1).

#### App User Interface

**Figure 2:**
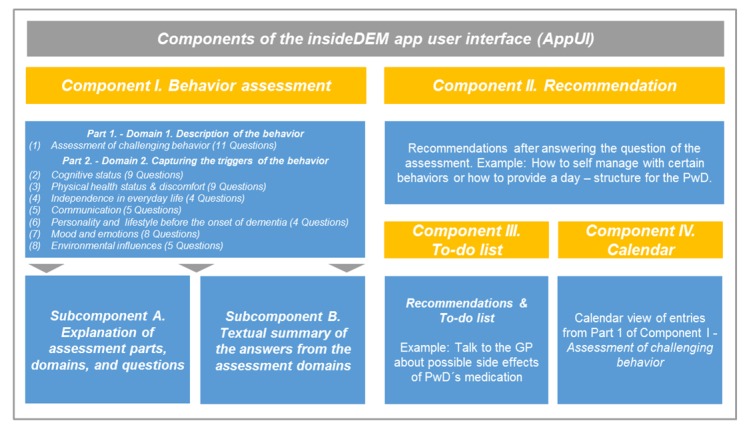
Components of the app user interface. GP: general practitioner; PwD: persons with dementia.

The key component of the ATI is an app user interface (AppUI) with the IdA [19], which was transformed into a digital app-based version. The AppUI consists of 4 major components (I-IV), shown in yellow in Figure 2.

##### Component I: Behavior Assessment

This component contains a home care–adapted digital version of the IdA that is divided into 2 parts with 8 domains encompassing 55 questions. Part 1: domain 1: description of the behavior includes 11 questions concerning general information on the behavior (description of the behavior, situation, frequency, occurrence, severity, and context) and the level of perceived burden [19]. Part 2: domains 2 to 8: capturing the triggers of the behavior: domain 2 cognitive status (9 questions), domain 3 physical health status and discomfort (9 questions), domain 4 independence in everyday life (4 questions), domain 5 communication (5 questions), domain 6 personality and lifestyle before the onset of dementia (4 questions), domain 7 mood and emotions (8 questions), and domain 8 environmental influences (5 questions).

In addition, component I includes 2 subcomponents: subcomponent A, explanation of assessment parts, domains, and questions and subcomponent B, textual summary of the collected information.

Subcomponent A is a guiding and educational element of AppUI intended to lead the caregiver through the assessment process (introduction to every assessment domain). Every question of the assessment is accompanied by an on-demand information button. This button will provide more detailed information about the specific topic of a question displayed on a pop-up screen. This information contains a textual explanation of why the specific question is important to answer in the context of CB, and there are examples of how caregivers obtain information to answer the question. In Subcomponent B, a textual summary of the information collected is shown after the user has completed an assessment domain. The collected data are slightly rephrased, and the summary has to be acknowledged by the user to ensure its validity.

##### Component II: Recommendation

On the basis of the collected information in domains 1 to 8 of component I, the user will obtain individualized recommendations for possible nonpharmacological interventions. For example, the assessment contains the question “Did you talk to the general practitioner (GP) about possible side effects of the medication?” Here, for example, the user obtains suggestions about important questions for the GP for the next visit.

##### Component III: To-Do List

The to-do list includes the important questions for the GP from the recommendations in component II. The list can be accessed by the user from the home screen.

##### Component IV: Calendar

The calendar shows all entries from the behavior assessment according to the date of the documentation from the user (component I).

#### Hardware Components and Technical Infrastructure

##### Home Server Operating Case

The delivery of the technical infrastructure is based on a modular and flexible distributed system architecture to provide a basis for future enhancements (eg, a more complex integration of sensor data). The hardware consists of a tablet personal computer and a sensor bracelet for gathering vital data from the PwD. The data processing (storage, distribution, and signal processing, which should be emphasized in future projects) is performed by a dedicated embedded system in the domicile of the study participants. A central home server is responsible for gathering data from all clients for observation and subsequent analysis and to enable further system extensions, for example, giving access to external entities such as caregivers and medical services. Moreover, this server is responsible for maintenance tasks and the automatic configuration and updating of the client systems (Figure 1). All involved systems use a message broker for communications in a secured environment (dedicated wireless local area network [WLAN] and virtual private network tunnels) and are accessible to external systems through standard interfaces. For the delivery of the technical infrastructure, we developed a home server operating case that includes the home server, an independent WLAN router and the capability for all devices to be charged by the user.

##### Sensor Bracelet

**Figure 3:**
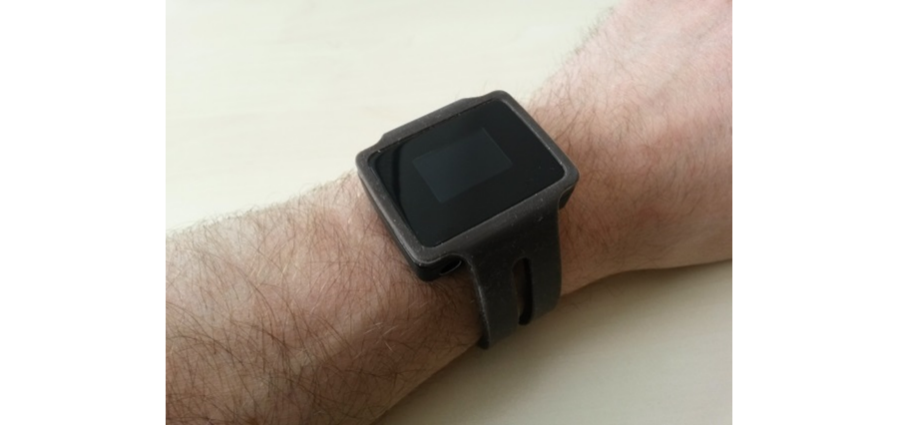
Sensor bracelet developed for insideDEM.

**Figure 4:**
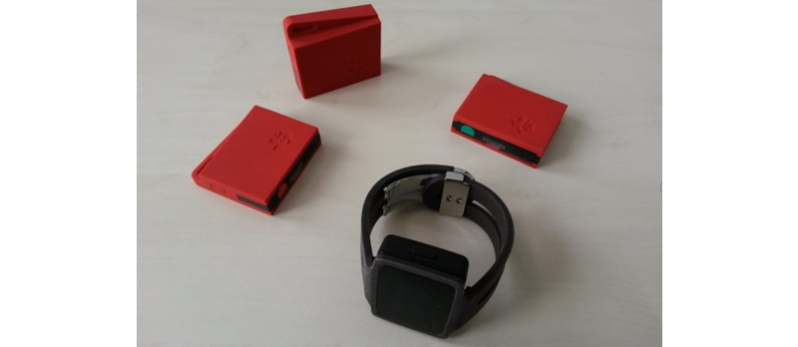
Bluetooth low energy beacon (red) and the sensor bracelet.

A sensor bracelet tailored to the specific needs of the insideDEM study has been developed [29,36]. As shown in Figure 3, this instrument is a watch-like device to be worn on the wrist or ankle. The bracelet contains numerous sensors to record data from the PwD and the environment. The sensors, including their specifications, are listed in Table 2. The bracelet is fully programmable and is currently setup in such a way that it records the data in a manner that is as detailed as possible. For example, accelerometers and gyroscopes record with a sampling rate of up to 100 Hz. On the basis of Bluetooth low-energy and dedicated beacons (Texas Instruments CC2650STK, shown in Figure 4), location information is recorded.

During the study, the bracelet has to be charged through a Universal Serial Bus connection to the home server. In this system, the *offload manager* is used to (1) load the recorded data from the bracelet, (2) scan the data with respect to symptoms indicating malfunctioning or an incorrect use of the sensor, and (3) prepare the bracelet for the next recording session (cleaning and synchronizing times). As soon as the sensor is ready for recording (indicated on the display) and detached from the home server, it starts recording the data, that is, no manual intervention is needed to prevent loss of data. The recorded data are transformed into an activity plot highlighting very active and very passive episodes. Due to the cognitive decline of the PwD, the primary caregiver of the PwD must be responsible for equipping the PwD with the nonintrusive sensor bracelet and for monitoring its proper functioning on a daily basis.

**Table 2:** Sensor modalities and corresponding sampling frequencies recorded by the sensor bracelet.

Sensor modality	Frequency of data
3-axis accelerometer	100 Hz
3-axis gyroscope	100 Hz
Skin temperature sensing	50 Hz
Reference temperature sensing	50 Hz
Photoplethysmography	50 Hz
Bluetooth beacon recording	On every Bluetooth event

The intervention assistants who deliver the ATI to the participants are trained nurses (later called intervention assistants) from a day care center of a communal residential care institution in Krefeld, Germany, with longstanding working experience in the care of PwDs. The intervention assistants are trained in using the ATI and in counseling alongside a self-developed delivery protocol (Multimedia Appendix 1). The caregivers obtain several in-house trainings (Table 3) and information sheets with important information about the ATI and the general study procedure. To manage participant attrition [37] and to encourage the use of the ATI, family dyads are visited twice after the first initial in-home visit. A second in-home visit is conducted in the second week and a third, in the fourth week with an intervention period at the end. In addition, intervention assistants provide the opportunity for individualized in-home visits, which can be requested by the participants through a telephone support hotline. The intervention assistants provide first-level troubleshooting for all technical problems. To provide a standardized process for all participants in the use of the ATI, in this early development phase, the participants are asked to complete the whole assessment in component I with all questions at least once in 1 week. In addition, the participants are encouraged to use the ATI as often as they feel comfortable doing so. From our previous study, we have found that most PwDs will have good compliance regarding the bracelet [29]. We are aware that wearing a device can be a burden for the PwD and it might not be tolerated. Especially during the first in-house face-to-face training for caregivers (1-1.5 hours), the intervention assistants will focus on how the bracelet will be tolerated by the PwD. If there is any sign of burden or extra CB by the PwD before or during the intervention, we will instruct the caregiver to take off the bracelet immediately. The ATI can be used without the bracelet.

### Delivery of Intervention

The process of delivery of the ATI is facilitated by different actions (Table 3).

**Table 3:** Components of the delivery of the intervention.

Timeline	Elements	Performance of the delivery
Preintervention	Training of the main intervention assistants in the use of the assistive technology (2 days and 10 hours)	Project team
Preintervention	Counseling training of the assistive technology intervention (2 days and 16 hours)	External provider
First week of intervention	In-house face-to-face training for caregivers (1-1.5 hours)	Intervention assistants
Second week of intervention	In-house visit and supervision of the caregivers	Intervention assistants
Fourth week of intervention	In-house visit and supervision of the caregivers	Intervention assistants
Fifth to eighth weeks of intervention	Additional in-house visits on demand	Intervention assistants
First to eighth weeks of intervention	Telephone hotline for prompt help, leaflet with written instructions	Intervention assistants

### Data Collection

#### Sociodemographic Data

The sociodemographic data of the PwD encompass gender, age, education, and year of diagnosis of dementia. The severity of the cognitive impairment is assessed according to the Global Deterioration Scale [38] at the baseline assessment before the intervention starts (T0) and after 8 weeks of the intervention (T1). The use of health care services is assessed with the questionnaire for Health-Related Resource Use in an Elderly Population (FIMA) [39]. This questionnaire includes 29 items focusing on aspects such as medication, GP visits, and other health care resources in the last 4 to 12 weeks.

For the primary caregiver, the sociodemographic characteristics include gender, age, education, living arrangement, hours of care per week, relationship to the PwD, and self-perceived stability of the care arrangement [40].

In addition to the sociodemographic data, the affinity for using technology of the family caregiver is assessed with the technology affinity questionnaire (TA-EG) at T0. In this questionnaire, affinity for using technology is defined as a personality characteristic that consists of trust in and a positive attitude and excitement toward the use of technologies (such as mobile phones and computers) [41]. The TA-EG involves 19 items rated with a 5-point Likert scale covering 4 domains: excitement related to technology use, self-perceived competence, perceived positive impact, and perceived negative impact of the use of technology [41]. A higher mean indicates a higher affinity for using technology [41].

#### Outcome Measures

Data on outcome measures are gathered face-to-face by trained interviewers (researchers of the German center for neurodegenerative diseases) with the family caregivers at T0 and T1 (Table 4). To provide maximum flexibility according to the individual needs of caregivers and PwDs, the interviews are conducted either at home or at the day care center that recruited the participants.

**Table 4:** Data collection for the outcome study.

Outcome or variable		Measurement	Number of items	Type of variable	Measurement
**Outcome for the person with dementia**					
	Challenging behavior	Neuropsychiatric inventory [35]	12	Outcome	T0-T1
	Agitation	Cohen-Mansfield agitation inventory [17]	29	Outcome	T0-T1
	Challenging behavior and agitation	Sensor data	Modalities according to Table 2	Outcome	Ongoing
**Outcome for caregivers of the person with dementia**					
	Skills to manage challenging behavior	Caregiver Assessment of Behavioral Skill Self-Report [47]	29	Outcome	T0-T1
	Quality of the current relationship	The Scale for the Quality of the Current Relationship in Caregiving [43]	14	Outcome	T0-T1
	Behavior related distress	Caregiver distress score from the Neuropsychiatric Inventory [35]	12	Outcome	T0-T1
	Self-perceived health	General Health Survey Questionnaire Short Form 12 [44]	12	Outcome	T0-T1
	Goals of caregiving	Goal Attainment Scale [46]	1	Outcome	T0-T1

### Person With Dementia

#### Challenging Behavior

The CB of the PwD is assessed with the NPI, proxy version [35]. The NPI assesses the presence, frequency, and severity of dementia-related behaviors in 12 different domains: delusions, hallucinations, depression, anxiety, euphoria, aggression, apathy, disinhibition, irritability, aberrant motor behavior, problems with sleeping, and appetite and eating disorders in the last 14 days. Frequency is rated on a 4-point scale (occasionally, often, frequently, and very frequently), and severity is rated on a 3-point scale (mild, moderate, and severe) [41]. The total NPI score will be calculated by adding the first 12 behavioral domains together. Therefore, we will calculate frequency×severity. A higher score indicates a higher level of the relevant domain of the NPI.

#### Agitation

For measurement of agitation, the CMAI [17] is used. The CMAI covers 29 items, each rated on a 7-point scale, to assess the occurrence and frequency of agitation (never, less than once a week but still occurring, once or twice a week, several times a week, once or twice a day, to several times a day, and several times an hour [17]). A higher cumulative score indicates a higher level of agitation.

### Family Caregiver

#### Skills to Manage Challenging Behavior

Self-reported management skills regarding CB from the perspective of the caregiver are measured with the German version of the Caregiver Assessment of Behavioral Skill Self-Report (CABS-SR). The CABS-SR includes 3 subscales: general approaches to caregiving (11 items), behavioral management of skill (17 items), and a single skill item scored between 1 and 4 as follows: 1=I do not do this very well; 2=I have some difficulty doing this; 3=I usually do this well; and 4=I do this very well. The cumulative score ranges between 11 and 44, with higher scores indicating higher levels of self-perceived skills [42].

#### The Quality of Current Relationship

The self-rated quality of the current relationship between the caregiver and the PwD is assessed with the scale for the Quality of the Current Relationship in Caregiving (QCPR), which includes 14 items scored on a 5-point scale (1: totally disagree; 2: disagree; 3: not sure; 4: agree; and 5: totally agree) [43]. The total score ranges from 14 to 70, with a median score more than 42 indicating a *better* relationship and less than 42 indicating a *poorer* relationship between the caregiver and the PwD [43].

#### Behavior-Related Distress

The behavior-related distress is assessed with the distress scale of the NPI. The distress is rated on a 5-point scale (no distress to minimal, mild, moderate, moderately severe, very severe, and extreme distress) [41]. The total distress score is generated by adding the scores of the 12 items from the questions related to distress [33].

#### Self-Perceived Health

Self-rated health is assessed with the General Health Survey Questionnaire Short Form 12 (SF-12) [44]. This instrument measures 8 different concepts such as physical functioning and role limitations because of general or physical health problems.

#### Goals of Caregiving

As standardized assessments often fail to depict the individual situation of complex care situations and the related problems [45], we measure the individual goal of caregivers on what should change in the care situation with the Goal Attainment Scale (GAS) [46], which has previously been used in dementia-specific technology studies [25]. At T0, the caregiver defines the specific goals that he or she would like to achieve using the AT. To indicate a subjective decrease or increase in the expected outcomes, numerical weights are assigned to evaluate goal attainment at T1: more than expected=1, much more than expected=2, less than expected=−1, and much less than expected=−2. Considering that behavioral or health-related aspects can change rapidly in a PwD, we ask the caregivers at T1 whether the goals are still relevant.

### Recruitment

A convenience sample of 20 dyads (primary caregiver-PwD) will be recruited face-to-face over a 5-month period. For pragmatic reasons, we determined the number of participants based on a realistic estimation of the intervention assistants in the day care centers. The intervention assistants from the day care center who are delivering the ATI to the home environment of the dyads are in charge of the recruitment process as well. In addition, the process of recruitment will be guided by the research team. The intervention assistants have longstanding working experience in the care of PwDs and a close relationship with the dyads to ensure the success of the recruitment. Different recruitment strategies used are (1) day care center with intervention assistants as gatekeepers, (2) a second local day care center as gatekeepers, (3) 2 neurologists as gatekeepers, and (4) an announcement in the local newspaper, followed by an open 2-hour information event at one of the day care centers. As the study will only take place in the home environment of the caregivers and the PwD, it is not important whether a PwD is a guest at the day care or not. In case of interest in the study, the intervention assistants conduct a face-to-face introduction with individuals and describe the aims, scope, study procedure, and participation requirements. Simultaneously, the potential participants receive written information about the study procedure and the document to give informed consent. After a minimum period of 7 days, the intervention assistants conduct a second detailed face-to-face introduction to receive the actual consent. Afterwards, the eligibility criteria are determined either in the care center or at the home of the families. No incentives are provided to participate in the study.

### Process Evaluation

**Figure 5:**
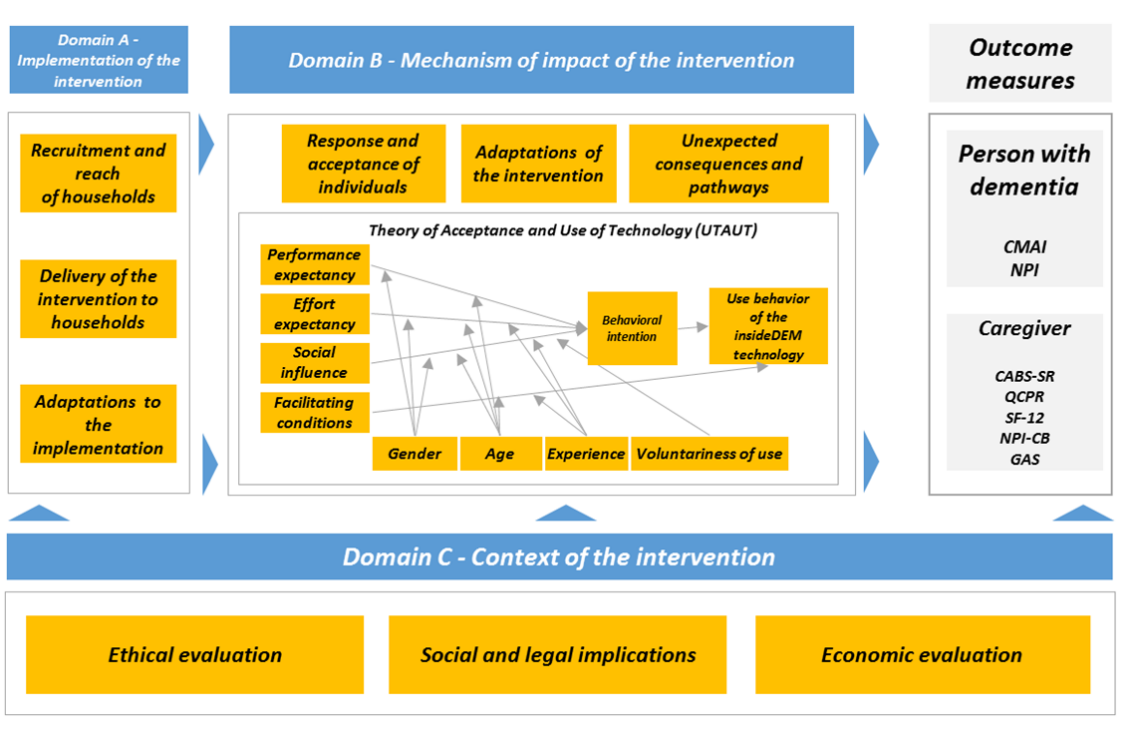
Framework of the insideDEM process evaluation. CABS-SR: Caregiver Assessment of Behavioral Skill Self-Report; CMAI: Cohen-Mansfield agitation inventory; GAS: Goal Attainment Scale; NPI: neuropsychiatric inventory; NPI-CB: neuropsychiatric inventory-challenging behavior; QCPR: Quality of the Current Relationship in Caregiving; SF-12: Short Form 12.

The domains of the process evaluation are guided by the MRC framework for the process evaluation of complex interventions [48]. The process evaluation addresses the following domains: (A) implementation of the intervention, (B) mechanism of impact of the intervention, and (C) context of the intervention. Each domain comprises different subdomains (Figure 5), for which different means of data collections are used.

#### Domain A: Implementation of the Intervention

This domain describes the process of recruitment and reach of households, the process of delivery of the intervention, and whether any adaptations were necessary according to what was initially planned in regard to how to implement the intervention. This aspect allows us to evaluate whether the implementation of the intervention was successful and how it possibly impacts the success of the intervention.

##### Subdomain: Recruitment and Reach of Households

The process of recruitment and reach is documented in a standardized handwritten recruitment protocol (Multimedia Appendix 1). As the intervention assistants are essentially in charge of the recruitment process, we additionally conduct semistructured qualitative interviews with them (Multimedia Appendix 1).

##### Subdomain: Delivery of the Intervention to Households

The intervention assistants document in a standardized handwritten delivery and intervention protocol whether all of the components of the intervention will actually be delivered to the participants (Multimedia Appendix 1). The protocol is applied during and after the initial in-house face-to-face training and is continued throughout the entire 8-week intervention period. Similarly, in this document, we record the feasibility of the application of the delivery curriculum. All nonconformities to the curriculum are documented by the intervention assistants. In addition, semistructured qualitative interviews are conducted with the intervention assistants to review the process of delivery (Multimedia Appendix 1).

##### Subdomain: Adaptations of the Implementation

Adaptations of the implementation of the intervention during the study phase are documented by the intervention assistants in a handwritten standardized delivery and intervention protocol (Multimedia Appendix 1). Furthermore, the intervention assistants will review the process of delivery after the initial delivery meeting on an audio recorder to obtain more qualitative data on the process of delivery (Multimedia Appendix 1). To monitor the quality of the delivery and to integrate potential adjustments immediately, a daily meeting among the researchers, intervention assistants, and developers of the software takes place in each week of the intervention phase. In addition, after the intervention, we will conduct semistructured qualitative interviews with the intervention assistants (Multimedia Appendix 1) and caregivers (Multimedia Appendix 1) to evaluate this domain.

#### Domain B: Mechanism of Impact of the Intervention

This domain describes the response and the user acceptance of the intervention, the unexpected consequences and pathways of the intervention, and the adaptations of the intervention.

##### Subdomain: Response and Acceptance

For the evaluation of the user acceptance, Venkatesh’s Unified Theory of Acceptance and Use of Technology (UTAUT) is used [49]. The UTAUT is a helpful model for analyzing technology acceptance in the field of dementia [50]. The UTAUT consists of 6 main variables: performance expectancy (PE), effort expectancy (EE), social influence (SI), facilitating conditions (FC), intention to use (ITU) and usage behavior (UB). PE is defined as the degree to which an individual believes that using the system will help or improve a certain task. EE is defined as the degree of ease that an individual associates with the use of the technology. SI is defined as the degree to which the user perceives that other important persons believe that the user should use the technology. FC is defined as the degree to which an organizational or technical infrastructure is available to support the use of the technology. In addition to acceptance, any unexpected consequences of using the AT are assessed. ITU is defined as “the degree to which a person has formulated conscious plans to perform or not perform some specified future behavior” [51], and UB describes the characteristics of use of the AT. The first 4 variables are moderated, in turn, by gender, age, experience, and voluntariness of use [49]. Information on the acceptance of the intervention from different perspectives is collected using quantitative and qualitative approaches. The Technology Usage Inventory (Multimedia Appendix 1) is a standardized questionnaire based on, for example, the UTAUT to evaluate the acceptance of new technology. It contains 30 items covering dimensions such as ITU, accessibility, user-friendliness, and usefulness. Higher scores in each domain indicate a higher level of acceptance. To assess system usability and the overall user experience and to adjust the user scenarios, we use the User Experience Questionnaire (UEQ) (Multimedia Appendix 1). The UEQ includes 26 pairs of opposite adjectives describing the attributes: attractiveness (6 pairs), perspicuity (4 pairs), efficiency (4 pairs), dependability (4 pairs), stimulation (4 pairs), and novelty (4 pairs); each pair is rated on a 7-point scale (from −3 to +3). A product with a highly rated usability is effective, efficient, and satisfying for the user and his or her needs. We administer the UEQ after the first use of the ATI and after the intervention period at T1. In addition, we assess the log files of the users’ app navigation and the overall UB characteristics via log files (Multimedia Appendix 1). The variables of interest are the time spent on a page, the time needed for the major tasks, the number of reverse navigations, the number of completed assessments (intervention component I), the number of documented behaviors per intervention period and per week (intervention component I), the number of displayed texts clicked per assessment question and the time spent on a specific text page (subcomponent A), and the number of user comments. Finally, we conduct qualitative semistructured interviews with the caregivers and intervention assistants based on the UTAUT (Multimedia Appendix 1), which will provide in-depth information on the reasons for using the ATI and further factors influencing its acceptance. To assess the acceptance of the ATI by the PwD, we use the caregiver as a proxy informant and the qualitative semistructured interviews with the intervention assistants (Multimedia Appendix 1). The duration of time the bracelet was actively worn is collected via log files (Multimedia Appendix 1).

##### Subdomain: Unexpected Consequences and Pathways

The semistructured interviews with the caregivers and the intervention assistants are analyzed regarding any unexpected consequences (Multimedia Appendix 1). Moreover, data from the delivery and intervention protocol as well as from the review the process of delivery are used (Multimedia Appendix 1).

##### Subdomain: Adaptations of the Intervention

With respect to the intervention’s adaptions, we distinguish between technology-external and technology-internal factors. Technology-internal aspects include bug fixes, periods of down time, and content changes during the intervention phase. Technology-external aspects include the counseling activities of the intervention assistants and the number of visits. The information source is the delivery and intervention protocol (Multimedia Appendix 1) and the semistructured qualitative interviews with the intervention assistants (Multimedia Appendix 1).

#### Domain C: Context of the Intervention

This domain describes any contextual factor that is external to the intervention and that potentially influences the impact of the intervention. In this study, we focus on ethical, social and legal, and economic implications (ELSIs).

##### Subdomain: Ethical Evaluation

The ethical evaluation is conducted in a workshop based on the model for the ethical evaluation for social-technical arrangements (MEESTAR) [52], including significant stakeholders, project partners, and members of the advisory board (Multimedia Appendix 1).

##### Subdomain: Social and Legal Implications

Expert interviews will be conducted to evaluate the social and legal implications and the perceived acceptance of the intervention in the field of home care (Multimedia Appendix 1). The experts represent different areas of the health care system such as medical device regulation (Medizinproduktegesetz), statutory health insurance companies, GPs, and home health care providers. As they play a major role in dementia care as gatekeepers for new technologies, we will conduct semistructured qualitative interviews with GPs and nurse managers of home health care providers in the home care setting (Multimedia Appendix 1). The aim is to assess their perspectives and attitudes regarding the use of the new ATI.

##### Subdomain: Economic Evaluation

The individual costs of the deployment, delivery, and standard operation of the ATI are calculated and described separately in an economic evaluation. Subsequently, a comparison of all originating costs is performed (Multimedia Appendix 1).

### Ethical Approval

The Ethics Committee of the German Society of Nursing Science approved the design and the study protocol in March 2017 (application number 17-004).

### Data Analysis

In this section, we describe the data analysis for the outcome evaluation and the process evaluation separately.

#### Outcome Evaluation

The outcome data are analyzed by applying descriptive statistics (means, SDs, and counts) relevant to the individual assessment. The Kolmogorov-Smirnov test is used to determine whether a sample is normally distributed [53]. After determining whether the related outcome samples of each assessment are normally distributed, we compare the 2 samples from T0 and T1 and analyze the differences between the 2 datasets. For non-normally distributed samples, we apply a Wilcoxon signed-rank test, and for normally distributed samples, a dependent-sample *t* test. Nominal data are compared with a chi-square test. For all quantitative data analyses, we use IBM SPSS Version 21. The significance level is set to 5%.

On the basis of the recorded data from the sensor bracelet, AMS [28] is computed; this score can be used to capture the overall activities of the PwD.

#### Process Evaluation

Descriptive statistics are applied to all quantitative data (Multimedia Appendix 1) and for all relevant log files (Multimedia Appendix 1). We compare the baseline characteristics of the quantitative data with the characteristics at T1.

All semistructured qualitative interviews (Multimedia Appendix 1) are transcribed into digital versions and subsequently analyzed by applying content analysis [54]. The handwritten recruitment protocol and the handwritten delivery and intervention protocol (Multimedia Appendix 1) are analyzed by using documentary analysis [55]. The results of the workshop based on the MEESTAR model are summarized in a workshop report (Multimedia Appendix 1).

## Results

The newly developed app- and sensor-based AT has been developed and was evaluated until July in 2018. The recruitment of dyads started in September 2017 and was concluded in March 2018. The data collection was completed at the end of July 2018.

## Discussion

The management of CB is a highly individual and complex task, and it poses a significant psychological and physical burden to the PwD and his or her caregivers [5]. To the best of our knowledge, the insideDEM technology is one of the very few examples to support the process of understanding the CB of the PwD via an ATI. In fact, to the best of our knowledge, there is no comparable technology that encompasses the functionalities of a caregiver assessment and a sensor assessment of the CB, questions that reflect possible factors influencing the CB, and the provision of recommendations to support caregivers to manage the CB of the PwD. In our view, it is important to evaluate the factors that shape the acceptance of the ATI from different perspectives as early as possible in the development of an ATI. This perspective is based on the assumption that acceptance is a necessary but not sufficient factor for evaluating the effectiveness of complex interventions [56]. The feasibility study will provide useful information on how to shape the intervention and the overall study procedure for trials at a larger scale. In the context of this study, understanding the delivery and use of the ATI in the real-life context of PwDs and their caregivers is indispensable.

A possible weakness of this study is that it is more likely that healthier and more motivated participants will take part in the study, which could possibly limit the results and transferability of the results for larger trials. A main concern is that the results could lead to an overestimation of the factors shaping the acceptance of the technology because of the participation of motivated and healthier participants. In addition, study attrition is a main concern, despite our strategy to mitigate this issue. Nevertheless, we think that the close and flexible support of the intervention assistants and their years-long experience in dementia care will lower this effect.

Before it is even possible to design a high-quality randomized controlled trial for this intervention, the process evaluation will provide valuable information for further steps of development by including the results of the intervention phase and the ELSIs. An important part of the ELSI criteria is the ethical aspects entailed in an ATI. Assistive systems may affect values such as independence or privacy and create tensions with other values such as safety or health. Moreover, different stakeholders hold different values, which further complicate the matter. Specifically designed for ATs, the model for the ethical evaluation of sociotechnical arrangements, MEESTAR [52], provides a suitable framework, allowing a normative ethical orientation in the design of an ATI.

## Appendix

Multimedia Appendix 1 Data sources of the process evaluation domains.

## Abbreviations

AMS: accelerometric motion score
AppUI: app user interface
AT: assistive technology
ATI: assistive technology intervention
CABS-SR: Caregiver Assessment of Behavioral Skill Self-Report
CB: challenging behavior
CMAI: Cohen-Mansfield agitation inventory
QCPR: Quality of the Current Relationship in Caregiving
EE: effort expectancy
ELSI: ethical, legal, and social implication
FC: facilitating conditions
GP: general practitioner
ICD: International Statistical Classification of Diseases and Related Health Problems
IdA: Innovative dementia-oriented Assessment system
ITU: intention to use
MEESTAR: model for the ethical evaluation for social-technical arrangements
MRC: Medical Research Council
NPI: neuropsychiatric inventory
PE: performance expectancy
PwD: persons with dementia
SF-12: Short Form 12
SI: social influence
TA-EG: technology affinity questionnaire
UB: usage behavior
UEQ: User Experience Questionnaire
UTAUT: Unified Theory of Acceptance and Use of Technology
WLAN: wireless local area network

## References

[1] Moniz Cook ED, Swift K, James I, Malouf R, De Vugt M, Verhey F (2012). Functional analysis-based interventions for challenging behaviour in dementia. Cochrane Database Syst Rev.

[2] James IA (2018). Understanding Behaviour in Dementia That Challenges: A Guide to Assessment and Treatment.

[3] Moniz-Cook E, Woods R, Gardiner E, Silver M, Agar S (2001). The Challenging Behaviour Scale (CBS): development of a scale for staff caring for older people in residential and nursing homes. Br J Clin Psychol.

[4] Moniz-Cook E, Hart C, Woods B, Whitaker C, James I, Russel I, Edwards RT, Hilton A, Orrell M, Campion P, Stokes G, Jones RS, Bird M, Poland F, Manthorpe J (2017). Challenge Demcare: management of challenging behaviour in dementia at home and in care homes – development, evaluation and implementation of an online individualised intervention for care homes; and a cohort study of specialist community mental health care for families.

[5] Feast A, Moniz-Cook E, Stoner C, Charlesworth G, Orrell M (2016). A systematic review of the relationship between behavioral and psychological symptoms (BPSD) and caregiver well-being. Int Psychogeriatr.

[6] de Vugt ME, Stevens F, Aalten P, Lousberg R, Jaspers N, Verhey FR (2005). A prospective study of the effects of behavioral symptoms on the institutionalization of patients with dementia. Int Psychogeriatr.

[7] Sansoni J, Anderson KH, Varona LM, Varela G (2013). Caregivers of Alzheimer's patients and factors influencing institutionalization of loved ones: some considerations on existing literature. Ann Ig.

[8] Wang J, Yu JT, Wang HF, Meng XF, Wang C, Tan CC, Tan L (2015). Pharmacological treatment of neuropsychiatric symptoms in Alzheimer's disease: a systematic review and meta-analysis. J Neurol Neurosurg Psychiatry.

[9] Ma H, Huang Y, Cong Z, Wang Y, Jiang W, Gao S, Zhu G (2014). The efficacy and safety of atypical antipsychotics for the treatment of dementia: a meta-analysis of randomized placebo-controlled trials. J Alzheimers Dis.

[10] Barton C, Ketelle R, Merrilees J, Miller B (2016). Non-pharmacological management of behavioral symptoms in frontotemporal and other dementias. Curr Neurol Neurosci Rep.

[11] Brodaty H, Arasaratnam C (2012). Meta-analysis of nonpharmacological interventions for neuropsychiatric symptoms of dementia. Am J Psychiatry.

[12] (2002). International Psychogeriatric Association.

[13] (2007). Dementia: The NICE-SCIE Guideline on Supporting People with Dementia and Their Carers in Health and Social Care (National Clinical Practice Guideline).

[14] Bartholomeyczik S, Halek M, Sowinski C, Besselmann K, Dürrmann P, Haupt M, Kuhn C, Müller-Hergl C, Perrar KM, Riesner C, Rüsing D, Schwerdt R, van der Kooij C, Zegelin A (2006). Gesamtpublikation Rahmenempfehlungen.

[15] Holle D, Halek M, Holle B, Pinkert C (2017). Individualized formulation-led interventions for analyzing and managing challenging behavior of people with dementia - an integrative review. Aging Ment Health.

[16] Cummings JL (1997). The Neuropsychiatric Inventory: assessing psychopathology in dementia patients. Neurology.

[17] Cohen-Mansfield J (1996). Assessment of agitation. Int Psychogeriatr.

[18] Teri L, Truax P, Logsdon R, Uomoto J, Zarit S, Vitaliano PP (1992). Assessment of behavioral problems in dementia: the revised memory and behavior problems checklist. Psychol Aging.

[19] Halek M, Holle D, Bartholomeyczik S (2017). Development and evaluation of the content validity, practicability and feasibility of the innovative dementia-oriented assessment system for challenging behaviour in residents with dementia. BMC Health Serv Res.

[20] Kolanowski AM, Whall AL (2000). Toward holistic theory-based intervention for dementia behavior. Holist Nurs Pract.

[21] Holle D, Krüger C, Halek M, Sirsch E, Bartholomeyczik S (2015). Experiences of nursing staff using dementia-specific case conferences in nursing homes. Am J Alzheimers Dis Other Demen.

[22] Parra-Vidales E, Soto-Pérez F, Perea-Bartolomé MV, Franco-Martín MA, Muñoz-Sánchez JL (2017). Online interventions for caregivers of people with dementia: a systematic review. Actas Esp Psiquiatr.

[23] Marshall M (1999). State Of The Art In Dementia Care.

[24] Khosravi P, Ghapanchi AH (2016). Investigating the effectiveness of technologies applied to assist seniors: a systematic literature review. Int J Med Inform.

[25] Kerssens C, Kumar R, Adams AE, Knott CC, Matalenas L, Sanford JA, Rogers WA (2015). Personalized technology to support older adults with and without cognitive impairment living at home. Am J Alzheimers Dis Other Demen.

[26] Lauriks S, Reinersmann A, Van der Roest HG, Meiland FJ, Davies RJ, Moelaert F, Mulvenna MD, Nugent CD, Dröes RM (2007). Review of ICT-based services for identified unmet needs in people with dementia. Ageing Res Rev.

[27] Meiland F, Innes A, Mountain G, Robinson L, van der Roest H, García-Casal JA, Gove D, Thyrian JR, Evans S, Dröes RM, Kelly F, Kurz A, Casey D, SzczeÅ›niak D, Dening T, Craven MP, Span M, Felzmann H, Tsolaki M, Franco-Martin M (2017). Technologies to support community-dwelling persons with dementia: a position paper on issues regarding development, usability, effectiveness and cost-effectiveness, deployment, and ethics. J Med Internet Res Rehabil Assist Technol.

[28] Kirste T, Hoffmeyer A, Koldrack P, Bauer A, Schubert S, Schröder S, Teipel S (2014). Detecting the effect of Alzheimer's disease on everyday motion behavior. J Alzheimers Dis.

[29] Teipel S, Heine C, Hein A, Krüger F, Kutschke A, Kernebeck S, Halek M, Bader S, Kirste T (2017). Multidimensional assessment of challenging behaviors in advanced stages of dementia in nursing homes-The insideDEM framework. Alzheimers Dement (Amst).

[30] Valembois L, Oasi C, Pariel S, Jarzebowski W, Lafuente-Lafuente C, Belmin J (2015). Wrist actigraphy: a simple way to record motor activity in elderly patients with dementia and apathy or aberrant motor behavior. J Nutr Health Aging.

[31] Kales HC, Gitlin LN, Stanislawski B, Marx K, Turnwald M, Watkins DC, Lyketsos CG (2017). WeCareAdvisor™: the development of a caregiver-focused, web-based program to assess and manage behavioral and psychological symptoms of dementia. Alzheimer Dis Assoc Disord.

[32] Craig P, Dieppe P, Macintyre S, Michie S, Nazareth I, Petticrew M, Medical Research Council Guidance (2008). Developing and evaluating complex interventions: the new Medical Research Council guidance. Br Med J.

[33] Campbell NC, Murray E, Darbyshire J, Emery J, Farmer A, Griffiths F, Guthrie B, Lester H, Wilson P, Kinmonth AL (2007). Designing and evaluating complex interventions to improve health care. Br Med J.

[34] Folstein MF, Folstein SE, McHugh PR (1975). "Mini-mental state". A practical method for grading the cognitive state of patients for the clinician. J Psychiatr Res.

[35] Cummings JL (1997). The Neuropsychiatric Inventory: assessing psychopathology in dementia patients. Neurology.

[36] Krüger F, Heine C, Bader S, Hein A, Teipel S, Kirste T (2017). On the applicability of clinical observation tools for human activity annotation. https://ieeexplore.ieee.org/document/7917545.

[37] Eysenbach G (2005). The law of attrition. J Med Internet Res.

[38] Reisberg B, Ferris SH, de Leon MJ, Crook T (1982). The Global Deterioration Scale for assessment of primary degenerative dementia. Am J Psychiatry.

[39] Seidl H, Bowles D, Bock JO, Brettschneider C, Greiner W, König HH, Holle R (2015). FIMA–Fragebogen zur erhebung von gesundheitsleistungen im alter: entwicklung und pilotstudie [FIMA--questionnaire for health-related resource use in an elderly population: development and pilot study]. Gesundheitswesen.

[40] von Kutzleben M, Köhler K, Dreyer J, Holle B, Roes M (2017). Stabilität von häuslichen Versorgungsarrangements für Menschen mit Demenz [in German]. Z Gerontol Geriatr.

[41] Kaufer DI, Cummings JL, Christine D, Bray T, Castellon S, Masterman D, MacMillan A, Ketchel P, DeKosky ST (1998). Assessing the impact of neuropsychiatric symptoms in Alzheimer's disease: the Neuropsychiatric Inventory Caregiver Distress Scale. J Am Geriatr Soc.

[42] Farran CJ, McCann JJ, Fogg LG, Etkin CD (2009). Developing a measurement strategy for assessing family caregiver skills: conceptual issues. Alzheimers care today.

[43] Spruytte N, Van Audenhove Chantal, Lammertyn F, Storms G (2002). The quality of the caregiving relationship in informal care for older adults with dementia and chronic psychiatric patients. Psychol Psychother.

[44] Ware J, Kosinski M, Keller SD (1996). A 12-item short-form health survey: construction of scales and preliminary tests of reliability and validity. Med Care.

[45] Hurn J, Kneebone I, Cropley M (2006). Goal setting as an outcome measure: a systematic review. Clin Rehabil.

[46] Schaefer I (2015). Universität Bielefeld - Fakultät für Gesundheitswissenschaften.

[47] Farran CJ, Fogg LG, McCann JJ, Etkin C, Dong X, Barnes LL (2011). Assessing family caregiver skill in managing behavioral symptoms of Alzheimer's disease. Aging Ment Health.

[48] Moore GF, Audrey S, Barker M, Bond L, Bonell C, Hardeman W, Moore L, O'Cathain A, Tinati T, Wight D, Baird J (2015). Process evaluation of complex interventions: Medical Research Council guidance. Br Med J.

[49] Venkatesh V, Morris MG, Davis GB, Davis FD (2003). User acceptance of information technology: toward a unified view. MIS Q.

[50] Liu L, Miguel Cruz A, Rios Rincon A, Buttar V, Ranson Q, Goertzen D (2015). What factors determine therapists' acceptance of new technologies for rehabilitation – a study using the Unified Theory of Acceptance and Use of Technology (UTAUT). Disabil Rehabil.

[51] de Veer AJ, Peeters JM, Brabers AE, Schellevis FG, Rademakers JJ, Francke AL (2015). Determinants of the intention to use e-Health by community dwelling older people. BMC Health Serv Res.

[52] Manzeschke A, Weber K, Rother H, Fangerau H (2013). Bundesministerium für Bildung und Forschung [Federal Ministry of Education and Research].

[53] Birnbaum ZW (1952). Numerical tabulation of the distribution of Kolmogorovs statistic for finite sample size. J Am Stat Assoc.

[54] Mayring P (2015). Qualitative Inhaltsanalyse: Grundlagen und Techniken.

[55] Coffey A, Flick U (2018). Analyzing Documents. The SAGE Handbook of Qualitative Data Analysis.

[56] Sekhon M, Cartwright M, Francis JJ (2017). Acceptability of healthcare interventions: an overview of reviews and development of a theoretical framework. BMC Health Serv Res.

