# Supplementary material for: A Tablet App– and Sensor-Based Assistive Technology Intervention for Informal Caregivers to Manage the Challenging Behavior of People With Dementia (the insideDEM Study): Protocol for a Feasibility Study
Source: JMIR Res Protoc. 2019 Feb 26;8(2):e11630. doi: 10.2196/11630 (PMC6412157; doi:10.2196/11630)
Supplement: Multimedia Appendix 1 [file resprot_v8i2e11630_app1.pdf]

Table 5 appendix

Table 5 Data sources of the process evaluation domains

Domain	Subdomain		Source of Data	Informant	Data Collection Procedure	Time of Data Collection
A. Implementation of the Intervention	Recruitment & Reach of Households	(a.)<br>(e.)	• Standardized handwritten recruitment protocol<br>• Interviews with intervention assistants based on UTAUT	• Intervention assistants	• Documented by intervention assistants<br>• Interview by trained interviewer (researcher of the DZNE)	• During the recruitment phase<br>• After the intervention
	Delivery to Households	(b.)<br>(c.)	• Standardized handwritten delivery and intervention protocol<br>• Audiotaped review of the delivery of the intervention	• Intervention assistants	• Documented by intervention assistants<br>• Audiotaped review by intervention assistants	• During the delivery<br>• After the delivery
	Adaptations of the Implementation	(b.)<br>(c.)<br>(d.)<br>(e.)	• Standardized handwritten delivery and intervention protocol<br>• Audiotaped review of the delivery of the intervention<br>• Interviews with intervention assistants based on UTAUT<br>• Audiotapes of semi-structured interviews with caregivers based on the UTAUT	• Intervention assistants<br>• Intervention assistants<br>• Intervention assistants<br>• Caregivers	• Documented by intervention assistants<br>• Audiotaped review by intervention assistants<br>• Interview by trained interviewer (researcher of the DZNE)<br>• Interview by trained interviewer (researcher of the DZNE)	• During and after the intervention
B. Mechanism of Impact of the Intervention	Acceptance	(f.)	• Technology Usage Inventory TUI	• Caregivers	• Standardized questionnaire (researcher of the DZNE)	• Before and after the intervention
		(g.)	• User Experience Questionnaire – UEQ	• Caregivers	• Standardized questionnaire	• After the first in-house-visit and after the intervention
		(h.)	• Log files	• System loggings (UI)	• System logging procedure	• Ongoing
		(d.)	• Audiotapes of semi-structured interviews with caregivers based on the UTAUT	• Caregivers	• Interview by trained interviewer (researcher of the DZNE)	• After the intervention
		(e.)	• Audiotapes of semi-structured interviews with intervention assistants based on the UTAUT	• Caregivers	• Interview by trained interviewer (researcher of the DZNE)	• After the intervention
	Unexpected Consequences and Pathways	(d.)<br>(e.)<br>(a.)	• Audiotapes of semi-structured interviews with caregivers based on the UTAUT<br>• Audiotapes of semi-structured interviews with intervention assistants based on the UTAUT<br>• Audiotaped review of the delivery of the intervention<br>• Standardized handwritten delivery and intervention protocol	• Caregivers<br>• Intervention assistants	• Interview by trained interviewer (researcher of the DZNE)<br>• Audiotaped review<br>• Documented by intervention assistants	After the intervention
	Adaptations of the intervention	(i.)	• Standardized handwritten delivery and intervention protocol<br>• Audiotapes of semi-structured interviews with intervention assistants based on the UTAUT	• Intervention assistants<br>• Caregivers	• Documented by intervention assistants (researcher of the DZNE)<br>• Interview by trained interviewer (researcher of the DZNE)	After the intervention
Conte xt of the	Ethical Evaluation	(m.)	• MEESTAR workshop report	• Study team	• Workshop with study team	After the intervention

Intervention	Social and Legal Implications	(n.)	• Audiotapes of semi-structured interviews with stakeholders, GPs and professional nurses based on UTAUT	• Stakeholders<br>• GPs and professional nurses	• Interview by trained interviewer (researcher of the DZNE)	Before and after the intervention
	Economic Evaluation	(o.)	• Report of the economic evaluation	• Researchers	-	Before, during and after the intervention
